# Community involvement facilitating the discussion of alcohol use in primary care: A nominal group study

**DOI:** 10.1080/13814788.2021.1936493

**Published:** 2021-06-24

**Authors:** Bram Pussig, Marc Van Nuland, Lodewijk Pas, Sarah Vandelanotte, Catharina Matheï, Bert Aertgeerts, Mieke Vermandere

**Affiliations:** a Department for Public Health and Primary Care, KU Leuven, Leuven, Belgium; b Department of Cardiovascular Sciences/Kulak, KU Leuven, Leuven, Belgium

**Keywords:** Qualitative research, community participation, harmful alcohol use, general practice, early identification and brief intervention

## Abstract

**Background:**

Hazardous alcohol use significantly affects health and wellbeing in society. General practitioners (GPs) are uniquely positioned to address this problem by integrating early identification and brief intervention (EIBI) in daily practice. Unfortunately, EIBI implementation remains low. Community-oriented strategies (COS), defined as public health activities directed to the general population, are suggested to address this implementation gap. COS aim to increase the understanding, engagement and empowerment within the population to facilitate EIBI delivery. However, no consensus on what COS should contain exists.

**Objectives:**

To obtain insight in the stakeholders’ perspectives and create consensus with them on COS with the highest potential to facilitate EIBI delivery.

**Methods:**

Four nominal group sessions were conducted with 31 stakeholders representing 12 different stakeholder groups from Leuven (Belgium). Stakeholders generated ideas, reflected on them in group and prioritised them anonymously, creating four separate lists. Merging these lists with their relative scores resulted in a master list, which was checked for accuracy through a member check. Qualitative content analysis on the stakeholder’s notes provided an in-depth exploration of their perspectives.

**Results:**

Twenty-one strategies were identified, nine of which were COS. Highlighting the GPs’ proactive role was considered most relevant. Other foci included creating awareness on the effects of alcohol use and normalising discussing alcohol use within the community. A holistic approach, exceeding the sole focus on COS, combining community, healthcare and government was accentuated.

**Conclusion:**

Stakeholders emphasise addressing the proactive role of GPs as most promising COS, though it should be delivered within a holistic multi-component approach.

KEY MESSAGES

- Community stakeholders provide practical, community-oriented guidance to facilitate the delivery of early identification and brief intervention.
- The community-oriented strategy with the highest potential comprises creating public awareness on the proactive role of general practitioners.
- Multi-sectoral collaboration, including the community, other healthcare providers and the local government, is essential for sustainability.

## Introduction

Drinking alcohol is essentially a social act embedded in many cultures, religions and communities [1], creating various situations that trigger alcohol consumption [2]. Nevertheless, alcohol remains one of the leading risk factors for morbidity and mortality in the world [3]. The healthcare burden even extends beyond the individual, as it also affects communities, families and entire health systems [4].

Early identification and brief intervention (EIBI) provides a means to reduce hazardous and harmful alcohol use in society. EIBI strives for optimal use of the primary healthcare setting to identify hazardous or harmful alcohol use early and provide straightforward advice when necessary [5]. This cost-effective strategy shows the highest efficacy when general practitioners (GPs) deliver it to non-treatment-seeking hazardous drinkers [6]. Unfortunately, implementation of EIBI remains low [7,8].

Initiatives on stimulating EIBI delivery in general practice have focussed on GP-related barriers [6]. These include limited time and lack of self-efficacy and legitimacy [9,10]. Even though these initiatives showed promising results, their overall effect sizes remained moderate [6,9]

Community-oriented strategies (COS) have been suggested to be essential for improving EIBI delivery in general practice [5]. COS are public health activities undertaken in the community [11]. A community is identified as a diverse group of people linked by social ties, shares common perspectives and engages with each other in a geographically defined location [12]. COS need to focus on reframing the public’s attitudes, norms, knowledge and views on alcohol to facilitate EIBI delivery in general practice [5]. Unfortunately, it remains challenging to determine what kind of strategies need to be undertaken to support GPs from a community perspective [13].

Integrating knowledge and experience within a community enables more ownership in a group of stakeholders, increasing the potential effectiveness of prevention strategies [14]. Therefore, efforts to implement EIBI must be customised to the local setting and emerge from the community itself [5,15]. This study aims to obtain an in-depth view of the stakeholders’ perspectives and create consensus within a community of stakeholders regarding related COS.

## Methods

### Study design

Four heterogeneous nominal group sessions were conducted simultaneously in May 2019. These nominal group sessions provide a means to generate ideas, solutions and priorities within a stakeholder population, while empowering all participants equally through structured group interactions [16]. The principles of the consolidated framework for reporting qualitative studies (COREQ) were followed [17].

### Participant selection and recruitment

This study was conducted in Leuven (Belgium), a medium-sized municipality with approximately 100,000 inhabitants and marked by a large student population [18]. Participating stakeholders had to live or practice their profession in Leuven.

Twelve relevant stakeholder groups, including laypeople, GPs, prevention workers, psychologists, communication experts, emergency doctors, pharmacists, mental health workers, social workers, dietitians, health insurance companies and law enforcement officers, were recruited (Appendix 1). A purposive sampling strategy was followed. Diverse recruitment strategies were applied, including flyers, e-mails, telephone calls, social media posts and personal contacts. Four nominal group sessions were composed in advance to ensure heterogeneity within the groups. This heterogeneity was inspired by combining scientific health promotion perspectives with those from the population of interest. The generated COS were thus both scientifically inspired and supported by the community itself. No extra sessions were planned due to data saturation, which was interpreted as the recurrence of comparable information between the groups [19].

### Data collection

Trained moderators, equipped with a written scenario and one observer, guided the groups through the phases of the nominal group technique. Each nominal group session comprised four phases: (1) generating ideas, (2) sharing ideas, (3) discussing ideas and (4) prioritising ideas (Appendix 2).

The moderator first explained the procedure and initiated an icebreaker discussion on the latest guidelines regarding the acceptable weekly intake of alcohol. Afterwards, participants were asked to discuss their views on barriers to raising alcohol use in general practice. Then, the main question was presented to the group: ‘*How can we facilitate the discussion on alcohol use with the general practitioner from a community-oriented perspective?’*

In the first idea-generating phase, participants could write down their initial thoughts. After twenty minutes, they were asked to share their ideas one by one. No interference from the other members was allowed during the sharing round. The moderator gathered all the ideas on a flip chart to maintain a structured progression in the conversations. Ideas were then clarified and grouped through group discussions. Afterwards, participants prioritised the clarified and grouped ideas individually by listing their motivated personal top five on paper.

### Data analysis

One researcher (B. P.) gathered all the data and constructed the four group-specific prioritised lists. These were based on the anonymous scoring forms provided by the participants. The lists were then analysed by three researchers (B. P., S. V., M. V. N.) and merged into a master list using a consensus meeting (Appendix 2). Ranking of the master list was constructed by calculating the relative scores (the absolute score divided by the group-specific number of participants) of all ideas within the separate groups. To evaluate the master list, a two-phased online member check was organised *via* Formsite (Vroman Systems, Downers Grove, IL). A member check comprises a feedback round with the participants after data analysis, improving the accuracy and credibility of qualitative findings [20]. The participants were asked to provide open-ended feedback on both the ranking of the ideas and the researchers’ reformulation of the individual strategies.

### Selection criteria for COS

The researchers formulated in- and exclusion criteria to determine which strategies of the master list also reflect COS. Suggested strategies were considered to be COS when they (1) directly address the general or patient population, (2) aim to lower barriers experienced in the general- or patient population for discussing alcohol use in general practice or if they (3) encourage the population to discuss alcohol use in general practice. One researcher (B. P.) reviewed the master list extracting the COS. Strategies that addressed the community indirectly and focussed on healthcare or government were excluded from the list and are presented in Appendix 3.

### Qualitative content analysis

The participants also provided a wealth of information in their notes. Therefore, these notes were also evaluated to gain a more in-depth view of the stakeholders’ perspectives. This was structured through qualitative content analysis [21]. Participants’ notes included personal notes made during the nominal group session, clarifications on scoring files and the observer’s notes. Three researchers (B. P., S. V., M. V. N.) performed this analysis.

## Results

### Participant recruitment

**Figure 1.**
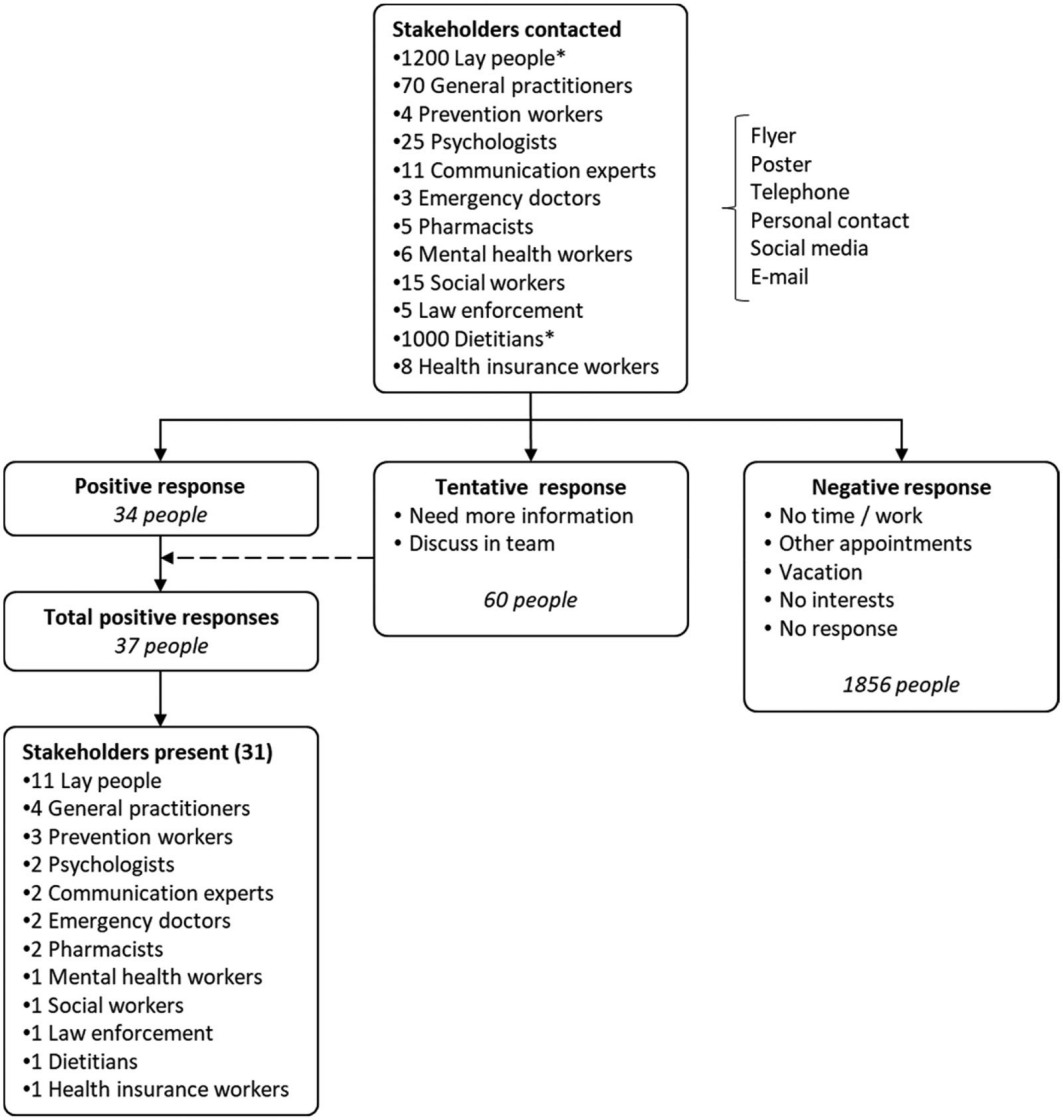
Overview of recruitment strategy (*) recruitment included flyers and social media, increasing the total amount reached.

Of the approximately 2350 people reached, 94 people showed interest, of whom 60 asked for more information to make their decision (Figure 1). Eventually, 31 stakeholders participated in the nominal group sessions, of whom 67.7% were women and 32.3% were men. The average age was 45 years, with age ranging from 26 to 81 years old. All groups were constructed in advance to establish a well-balanced representation of the different stakeholder groups (Table 1). Eighty per cent of the participants provided feedback during the member check.

**Table 1.** Demographics and nominal group compositions.

	Group 1	Group 2	Group 3	Group 4
Men	50%	25%	25%	57%
Women	50%	75%	75%	43%
Composition				
General population	2	3	3	3
General practitioner	1	2	1	
Psychologist	1	1		
Prevention worker	1		1	1
Emergency doctor		1	1	
Communication expert	1		1	
Mental health worker	1			
Social worker	1			
Dietitian				1
Pharmacist		1		1
Law enforcement			1	
Health insurance company				1
Attendance		Pharmacist and 1 GP left early		

GP: general practitioner.

### Nominal group technique and member check

The four nominal group sessions produced 58 ideas or strategies to facilitate the discussion on alcohol use with GPs. After merging the 4 separate lists of strategies into a master list and completing the member check, 21 items remained (Appendix 3). Nine items were considered COS that directly address the population (Table 2). The other ideas address the community indirectly by focussing on healthcare professionals (*n* = 8) or governmental regulatory bodies (*n* = 4) (Appendix 3). Creating awareness on the proactive role of GPs was the most prominent strategy of those listed with a view on addressing the community directly.

**Table 2.** Stakeholders approved master list with community-oriented strategies to facilitate the delivery of alcohol-related EIBI in general practice from a stakeholder point of view.

Idea / Strategy	Overarching relative score^a^
Awareness campaign directed to the general population highlighting the pro-active role of the GP concerning alcohol use, alcohol-related questions and health promotion	1151
Media campaign (e.g. posters, flyers, videos) related to alcohol in the waiting room of GPs to spike the patient’s interest; possible conversation starter	579
Formal (the news, talk shows) and informal (soaps) media attention focussing on the effects of harmful alcohol use with a link regarding the GP as a reliable source of information	554
Initiating conversations on alcohol use in community specific settings, stepping outside the areas of healthcare and general practice	453
Informative campaign towards the general population concerning the effects of (harmful) alcohol use	426
Social norm campaign in the community	393
Mobile or web-based self-awareness tool to assess one’s own consumption pattern	338
A ‘silent alarm’ (e.g. a notification) for community members to inform their GP about the need to discuss alcohol related topics	138
Structural interventions to highlight the current social norm on alcohol	113

^a^Group-specific scores were divided by the participant count of that group. Summation of the relative scores from the four groups resulted in the overarching relative score. GP: general practitioner.

Qualitative content analysis, which combined the master list (Appendix 3) and the field notes, allowed a more in-depth depiction of stakeholders’ insights. During this analysis, three distinct foci arose.

### Community-oriented strategies

All participants agreed that creating awareness in the community is crucial to evolve to a more favourable situation for delivering alcohol-related EIBI in general practice. Further, the groups extensively discussed that multiple underlying issues would need to be addressed to create a compelling and sustainable approach. Participants mentioned the community’s lack of knowledge on detrimental health effects of alcohol use, current social norms and stigma regarding alcohol use. The stakeholders pointed out that a lack of knowledge on the differences between harmful alcohol use, hazardous alcohol use and alcohol dependence might facilitate stigma within the community. In an attempt to address these barriers, participants’ notes indicated a two-phased approach for designing COS.

### Phase I

The first phase comprises the necessity to determine the needs and concerns of the community to facilitate the discussion of alcohol use in general practice. Considerable attention was given to knowledge transfer related to the physical, mental and social effects of alcohol, emphasising social and familial contexts.

### Phase II

The second phase revolves around how these goals could be accomplished. Most of the ideas leaned towards mass media campaigns using multiple strategies to convey different messages. Materials to be used should include flyers, posters, commercials (on television, radio and in cinemas), papers, magazines, social media posts and infographics. Furthermore, two groups discussed the use of self-awareness tools. The threshold for directly discussing alcohol with a GP was considered too large and self-screening could be helpful to overcome this barrier.

Next, it was suggested that the discussion on alcohol use in general practice could be normalised by initiating the conversation outside general practice. Pharmacists, schools, neighbourhood centres and other relevant community organisations were mentioned as possible facilitators. This approach might increase the self-legitimacy of GPs and lower the barrier for discussing alcohol in the general population. Finally, ideas of creating an overall more supportive environment by making alternatives to alcohol more appealing and making it harder to drink excessively were discussed.

Participants also stressed the importance of diversifying the means of communication and the messages conveyed. Diversification allows addressing specific population groups (including multiculturality, age and gender). Existing materials and campaigns should be re-examined to integrate new insights. Positive messaging should be used in all communications without minimising the problem. Appealing campaigns should be combined with coordinated and formal communication on the effects of alcohol use (e.g. talk shows, flyers, posters).

### GP-related strategies

Participants stressed the crucial role of GPs in alcohol-related EIBI uptake. They indicated that they are willing to discuss personal alcohol use with their GP. However, more skills training for GPs on EIBI delivery (e.g. motivational interviewing and communication skills) was stressed. Participants discussed the use of a formal recognition for GPs specialised in alcohol-related consultations. Additionally, collaborative care was thought to be necessary. Participants suggested introducing a comprehensive database allowing various healthcare professionals to share relevant patient data (e.g. related to alcohol). Participants believed that this would improve the communication between healthcare professionals, enabling a uniform health promotion approach. Furthermore, there is a need to create consensus on a referral strategy and task description to support healthcare professionals in adequately helping patients when brief advice is insufficient.

### Government-related strategies

Participants focussed on the importance of governmental support to create sustainable public health initiatives. According to them, there is a need to change the financial model for healthcare professionals to participate more in preventive medicine. It was suggested that there should be free alcohol consultations where people can address their alcohol-related concerns.

Next, participants highlighted the need for governments to invest more in stricter regulatory measures, such as stricter regulations on labelling, commercials, outlet density, taxation as well as increasing the age limit for drinking alcohol.

All participants agreed that a holistic approach combining efforts by the community, healthcare professionals and government is essential for sustainability. Participants emphasised the potential synergy between strategies.

## Discussion

### Main findings

A comprehensive list with nine COS emerged as a result of this study. This list provides an extensive view of COS with the highest potential to lower community-related barriers for discussing alcohol use in general practice and facilitate EIBI delivery. These strategies are aimed to increase awareness, knowledge and health literacy within the general population regarding alcohol use and the role of GPs. Further, the stakeholders highlighted the need to tackle the social stigma and norms towards alcohol use. The top-ranked strategies focus first and foremost on creating awareness within the community and emphasising the proactive role of GPs to perform alcohol-related health promotion. During the stakeholder discussions, other foci arose targeting healthcare professionals and governmental organisations. Community stakeholders acknowledge the importance of a holistic approach to create an effective and sustainable concept facilitating EIBI integration in general practice.

### Strengths and limitations

A well-established methodological design was used to ensure scientifically supported proceedings and minimising potential bias [16,22]. Experienced moderators were extensively briefed to allow a uniform progression between the nominal group sessions.

The heterogeneous composition of the nominal group sessions provided a unique interplay among participants, resulting in interesting group discussions and comprehensive field notes. Based on the qualitative content analysis, elements of COS emerged. They provide a means of structuring COS development and address the necessity of combining community, healthcare and government. This analysis explored elements that are not necessarily community-specific. They highlight broader strategies such as using mass media campaigns or facilitating the discussion on alcohol use outside the clinical setting. To our knowledge, this is the first study that created consensus on COS, accentuating potential effectiveness in facilitating EIBI delivery general practice.

The number of participating GPs and social workers was lower than anticipated, mostly due to time constraints. Their relatively lower number might have affected the output because they both have a prominent role in the community.

### Comparison with existing literature

Mass media campaigns have shown to effectively change alcohol-related attitudes, beliefs and knowledge [9,23]. There is, however, still some uncertainty regarding their effectiveness in changing behaviour itself [23]. Mass media has been suggested as a means to increase EIBI delivery in general practice [5]. However, to our knowledge, no empirical evidence supporting this hypothesis exists [24]. Furthermore, mass media campaigns with specific community-integrated actions increase the effectiveness of changing behaviour [25]. This supports participants’ belief that the strategies discussed do not only function separately, but that there might be some synergetic effects.

Alcohol-related self-awareness tools have shown to effectively stimulate the discussion on alcohol use with healthcare professionals in an adolescent population [26]. Moreover, digitalised self-assessment tools are shown to be more effective when compared to an on-paper alternative presented in waiting rooms [26]. Interestingly, the participants in our study suggested using mobile or web-based self-awareness tools. Such tools are also known to be accepted when implemented in non-clinical settings [27]. However, it is unclear if this approach also has the same effectiveness compared to presenting self-awareness tools in the GP’s waiting rooms [27].

The other suggestions reflecting COS here are newly presented as possible interventions to facilitate alcohol-related EIBI in general practice. These include the need for initiating the conversation of alcohol use outside the clinical setting, a silent alarm to inform GPs and structural interventions highlighting the norm on alcohol.

The proposed necessity of combining community, healthcare professionals and governmental organisations to create an effective and sustainable project seems to be a generic and recurrent concept [24]. The observation of this holistic approach may not have been the primary objective of our study. However, it might trigger all parties to undertake action, making a real difference in reducing harmful alcohol use in society.

### Implications for general practice

Strategies addressing population-related barriers are believed to lower the threshold to initiate the discussion of alcohol use in general practice. When implemented, these strategies might also positively affect GPs’ legitimacy, time management and eventually their self-adequacy for delivering EIBI. The decision on which COS would be most effective within a community depends on local preferences, financial support and the community’s needs. It is clear that a holistic approach, combining different strategies, will be more effective for a sustainable implementation, fading the barriers for EIBI implementation.

In the long term, we aim to trigger policymakers, healthcare professionals and the general population to undertake combined initiatives in their local communities. This could encourage these different stakeholder groups to join forces creating a durable and trustworthy situation in which GPs and the general population feel comfortable working together towards a healthier society.

## Conclusion

Community stakeholders provided new insights on COS with the highest potential to facilitate EIBI delivery in general practice. These strategies are believed to reinforce previous attempts to increase EIBI delivery by addressing the implementation gap from a new perspective, i.e. that of the community.

## Appendix 1. Overview of the purposeful sampling reasoning

**Table:**

Stakeholder group	Purpose for inclusion
General practitioner	As the aim of this study is to create consensus on community-oriented strategies to support GPs in de delivery of EIBI it is essential to incorporate their point of view and their needs or concerns.
Lay people	They represent the population of interest to address with the community-oriented strategies. The Lay people provide insights in community perspective.
Social worker	Social workers have a strong connection with the community, especially with a proportion that is generally more difficult to reach (ethnicity, social economic status, *et cetera*).
Prevention worker	They are part of a governmental organisation responsible for local preventive work in the community. Prevention workers have a good idea of the possibilities in the municipality. They can give more insight in what might work at municipal level.
Psychologist	Psychologists provide a scientific point of view on behaviour change techniques.
Communication expert	They have a better understanding on the scientific perspective on how to communicate sensitive topics like alcohol to the general population. Furthermore, they provide insight in how to facilitate behavioural change trough communication.
Pharmacist	They come in contact with a large part of the general population. They also have an increasing potential to be involved in prevention approaches, especially when it related to alcohol use.
Dietitian	Has a unique position to ask about alcohol use and health. Furthermore, dietitians are increasingly involved in health promotion and prevention.
Mental health worker	Mental health workers bring the other spectrum of alcohol use to the discussion. They allow to address the seriousness of harmful alcohol use to the less experienced participants. In addition, they are able to address the topic from another angle.
Health insurance company	Besides providing the population with health insurances, health insurance companies also undertake a significant amount of health promotion and prevention activities. They provide insight from another perspective.
Law enforcement	The police has a strong connection with the local community. Their role in prevention is often undervalued. However, they come in contact with a large proportion of the population and experience the local necessities first-hand.
Emergency doctor	The emergency department comes in contact with a lot of harmful and hazardous alcohol use. They have the opportunity and willingness to participate in prevention work, especially when it is alcohol related.

## Appendix 2. Visualisation of the nominal group and member check progression

**The nominal group progression. Each nominal group session comprised four phases:**

**Merging of the nominal groups’ results in a master list and the online member check**

## Appendix 3. Stakeholder approved master list with all the ideas discussed by the participants

**Table:**

Idea/strategy	Overarching relative score^a^
Awareness campaign directed to the general population highlighting the pro-active role of the GP concerning alcohol use, alcohol-related questions and health promotion	1151
Media campaign (e.g. posters, flyers, videos) related to alcohol in the waiting room of GPs to spike the patient’s interest; possible conversation starter	579
Formal (the news, talk shows) and informal (soaps) media attention focussing on the effects of harmful alcohol use with a link regarding the GP as a reliable source of information	554
Initiating conversations on alcohol use in community specific settings, stepping outside the areas of healthcare and general practice	453
Skills training for GPs regarding behaviour change strategies and communication skills	435
Informative campaign towards the general population concerning the effects of (harmful) alcohol use	426
Social norm campaign in the community	393
Mobile or web-based self-awareness tool to assess one’s own consumption pattern	338
More initiative should come from the GPs to start the conversation on alcohol	175
Integrating other healthcare professionals besides GPs into the early identification process, stepping outside of general practice	162
A ‘silent alarm’ (e.g. a notification) for community members to inform their GP about the need to discuss alcohol related topics	138
Initiating free alcohol-consults to discuss alcohol with the GP	114
Structural interventions to highlight the current social norm on alcohol	113
Introducing a financial model which stimulates GPs to do more pro-active work	92
Integrating more psychologists in general practice, thus, reducing waiting lists and stimulating collaboration for a better follow-up	88
Government support for educating the population on the dangers of alcohol use	86
Establishing a database containing patient information related to alcohol for use by all healthcare professionals, thus increasing the likelihood of early identification	71
Introducing standard yearly screenings for alcohol by the GP	67
A recognition for GPs with a specialisation to guide people with alcohol related questions and issues	63
Reorienting the focus to the underlying issues responsible for causing the harmful use of alcohol (mental health focus); integrating mental health workers	50
Mandatory ‘alcohol-consultations’ with a GP after repeated encounters with law enforcement related to alcohol use	50

^a^Group-specific scores divided by the participant count of that group. Summation of the relative scores from the four groups resulted in the overarching relative score. GP: General Practitioner.
